# Mothers' perceptions of child weight status and the subsequent weight gain of their children: a population-based longitudinal study

**DOI:** 10.1038/ijo.2017.20

**Published:** 2017-02-21

**Authors:** K N Parkinson, J J Reilly, L Basterfield, J K Reilly, X Janssen, A R Jones, L R Cutler, A Le Couteur, A J Adamson

**Affiliations:** 1 Institute of Health and Society, Newcastle University, Newcastle upon Tyne, UK; 2 Human Nutrition Research Centre, Newcastle University, Newcastle upon Tyne, UK; 3 Physical Activity for Health Group, School of Psychological Sciences and Health, University of Strathclyde, Glasgow, UK; 4 Northumberland, Tyne and Wear NHS Foundation Trust, Newcastle upon Tyne, UK

## Abstract

**Background:**

There is a plethora of cross-sectional work on maternal perceptions of child weight status showing that mothers typically do not classify their overweight child as being overweight according to commonly used clinical criteria. Awareness of overweight in their child is regarded as an important prerequisite for mothers to initiate appropriate action. The gap in the literature is determining whether, if mothers do classify their overweight child's weight status correctly, this is associated with a positive outcome for the child's body mass index (BMI) at a later stage.

**Objective:**

To explore longitudinal perceptions of child weight status from mothers of a contemporary population-based birth cohort (Gateshead Millennium Study) and relationships of these perceptions with future child weight gain.

**Methods:**

Data collected in the same cohort at 7, 12 and 15 years of age: mothers' responses to two items concerning their child's body size; child's and mother's BMI; pubertal maturation; demographic information.

**Results:**

Mothers' perceptions of whether their child was overweight did not change markedly over time. Child BMI was the only significant predictor of mothers' classification of overweight status, and it was only at the extreme end of the overweight range and in the obese range that mothers reliably described their child as overweight. Even when mothers did appropriately classify their child as overweight at an earlier stage, this was not related to relatively lower child BMI a few years later.

**Conclusions:**

Mothers tend to classify their child as overweight in only more extreme cases. It is an important finding that no beneficial impact was shown on later child BMI in overweight children whose mothers classified their child's weight status as overweight at an earlier stage.

## Introduction

Over 30% of children in the United Kingdom (UK) are overweight or obese^1^ based on the UK national body mass index (BMI) centiles classification of being at or above the 85th and 95th centiles, respectively.^2^ This is a major public health concern because of the physical and psychological impact on the child.^3^ There is considerable evidence that parents typically do not classify their child as overweight or obese even when their child is according to commonly used clinical criteria.^4, 5^ Parents report comparing their child's weight to that of other children,^6^ and as childhood obesity rates increase, parents may compare their child to increasingly overweight children, resulting in a shift in social norms related to body weight.^7^

Our own previous work on this topic in English children from the Gateshead Millennium Study population-based birth cohort showed that at age 7 years, the children typically needed to be in the obese range to be classified as overweight by their mother.^8^ However, parents did underestimate the weight status of overweight children less as BMI increased,^8^ consistent with previous findings.^4^ This demonstrates that parents are aware of overweight in their child but are not sensitive to its lower levels.

Parents' ability to classify accurately their child's weight status is regarded as important. For example, underestimation is claimed to be a barrier for behaviour change,^9^ and the ability of mothers to recognise when their child is becoming overweight is claimed to be key to childhood obesity prevention.^10^ These assertions were not evidence based. Whether mothers are concerned about their child becoming overweight in the future is also important—it is perhaps something all parents should be concerned about, but particularly so if their child is already overweight as we know that overweight in childhood tends to ‘track' into adulthood.^11^

The majority of studies of parental perceptions of childhood overweight and obesity are cross-sectional studies of mothers of children up to 12 years^12, 13^ who were recruited predominantly from schools or health-care facilities.^12^ To our knowledge, there are three longitudinal studies published on repeated measures of mothers' perceptions of their child's body weight.^14, 15, 16^ In one, the study reports on a UK sample that mothers' correct classification of their child's weight status improved from 44% at 7 years to 74% at 16 years, with child BMI predicting correct classification at both time points. There was no evidence that correct classification of the child being overweight at 7 years impacted on the trajectory of the child's BMI from 7 to 16 years.^15^ Both the other papers (one in a small non-representative sample of 5–9-year-old children in the Netherlands;^17^ one in a nationally representative Australian sample of 4–13-year-old children^14^) report that parental identification of child overweight was associated with more weight gain at follow-up. Taken together, the evidence from these studies challenges the assumption that accurate classification of child weight status is important for appropriate parental action. It is important that further similar studies are conducted to check for replicability or otherwise,^18^ to determine whether there is supporting evidence for this assumption.

The present study investigated longitudinal data on mothers' classification of their child's weight status in a population-based birth cohort. Using data from three follow-ups (at ages 7, 12 and 15 years), the study had three aims. The first aim was to describe and examine the mother's classification of her child's weight status and whether she had any concerns about her child becoming overweight in the future. The second aim was to quantify the BMI at which mothers typically classify their child as overweight, and at which they express concern about their child becoming overweight in the future. The third aim was to investigate whether any specific child characteristics (sex, BMI and pubertal completion) and/or maternal characteristics (BMI and socioeconomic status) are associated with correct classification of child weight status.

## Materials and methods

### Participants

The data reported are from the longitudinal Gateshead Millennium Study.^19^ All infants born to mothers resident in Gateshead, northeast England, in prespecified weeks in 1999/2000 were eligible. In all, 1029 infants (82%) were recruited shortly after birth.^20^ Mothers were primarily from the white ethnic majority group (98%).^19^ Full details are published.^19, 20^ At each follow-up, all the families that have not previously asked to leave the study are eligible to participate. This paper reports on questionnaire and body measurement data when the children were 6–8 years (median 7, referred to as 7 years in this paper), 11–13 years (median 12, referred to as 12 years) and 15–16 years (median 15, referred to as 15 years).

Favourable ethical opinions were granted by Gateshead and South Tyneside Local Research Ethics Committee (7 years: reference 06/Q0901/49) and by Newcastle University Ethics Committee (12 and 15 years: references 00510/2011 and 00728/2014). Parents gave written consent for their own and their child's participation in the study. Children gave written assent (7 and 12 years) and consent (15 years) for their participation.

### Procedure

At the 7-year follow-up, trained researchers visited the mothers at their home to administer the 7-year questionnaire and take their body measurements. At the 12- and 15-year follow-ups, mothers were sent the 12- and 15-year questionnaire, respectively, and asked to return the completed questionnaire by post. At all three follow-ups, the children were visited at school or home to take their body measurements.

### Measures

At each follow-up, the parental questionnaire included two items from a previous study:^21^ ‘How would you describe your child's weight at the moment?' (very underweight; underweight; normal; overweight; very overweight) and ‘How concerned are you about your child becoming overweight in the future?' (unconcerned; a little concerned; concerned; fairly concerned; very concerned).

The child questionnaire administered at 12 and 15 years included the Pubertal Development Scale (PDS), a self-report measure of pubertal status for young adolescents with good reliability and validity.^22^ The scale was adapted for the 15-year follow-up to include the age the pubertal events began and were completed.

Body measurements were taken by the researchers at the 7-year follow-up for the mothers and at each follow-up for the children:

- (i) Height measured to 0.1 cm with the head in the Frankfurt plane using a Leicester portable height measure.
- (ii) Weight measured to 0.1 kg using TANITA scales TBF 300MA (TANITA Corporation, Tokyo, Japan).

On each assessment occasion, measurements were taken in duplicate or more if necessary until two values were obtained within 1.0 cm of each other for height and within 0.1 kg of each other for weight.

Details about the family including socioeconomic status measures were collected at recruitment, shortly after birth. From this, the family's postcode transformed into the Townsend deprivation score.^23^ The Townsend deprivation score was used to assess the representativeness of the participating cohort after attrition since recruitment, and as a potential confounder variable in the main analyses.

### Statistical analysis

Mothers' responses to the item about their child's current weight status were dichotomised into a ‘not overweight' category (very underweight, underweight or normal weight) versus an ‘overweight' category (overweight or very overweight).

The mean of the duplicate weight and height measurements were used to calculate BMI (weight (kg)/height (m)^2^) in the mothers and children. For children, the UK 1990 BMI reference curves were used,^2^ which defines BMI ⩾91st and ⩾98th centiles as overweight and obese, respectively. The centiles were calculated using lmsGrowth.^23^ As our interest in this study was in identification of overweight, at each age the children were dichotomised into ‘normal weight' and ‘overweight' the latter group included obese children.

Using the binary variables described above derived from the mothers' responses to the questionnaire item and the child's weight status according to the UK 1990 BMI reference curves,^2^ a further dichotomous variable was created to categorise mothers as ‘correct' or ‘incorrect' in the classification of their child's weight status.

Mothers' responses to the questionnaire item about whether they were concerned about their child becoming overweight in the future were dichotomised into the ‘unconcerned' category versus a ‘concerned' category (a little concerned, concerned, fairly concerned, very concerned).

Using the data from both follow-ups, a categorised measure of the time of pubertal maturation was created based on the classification now recommended by the Royal College of Paediatrics and Child Health.^24^ For girls these were: early (up to 12 years), mid (12 years) and late (13 years and over). For boys these were: early (up to 13 years), mid (13 and 14 years) and late (15 years and over).

To ensure statistical independence one twin from each pair was dropped at random. Cross-sectional analyses were restricted to children for whom the mother was the respondent with concurrent data for both questionnaire items and child BMI data. For longitudinal analysis, selection of cases was listwise. Logistic regression was used to examine relationships between the mothers' dichotomised variables and the predictor variables. Linear regression was used to examine predictors of child BMI. Two-tailed statistical tests are reported.

SPSS version 21 (SPSS Inc., Chicago, IL, USA) was used for statistical analysis.

## Results

Using the selection criteria described, data were available from 545 children at 7 years, 443 at 12 years and 305 at 15 years. One twin from each pair was dropped at random (14 at 7 years; 10 at 12 years; 7 at 15 years) providing samples of 531 at 7 years, 433 at 12 years and 298 at 15 years. The longitudinal analyses for which data from mothers at all three follow-ups were available was based on a sample of 228 children.

### Attrition from the original cohort

Attrition was assessed by the Townsend deprivation index from the UK 1991 census (Table 1). The original sample was comparable with the northeast region of England in terms of socioeconomic deprivation apart from slight under-representation of the most affluent quintile. Overall, the distribution across all the quintiles at each time point is fairly even, although attrition has been higher over time in the lower quintiles.

We tested for differences in BMI at 7 years between participants and non-participants at 12 years (*t*=1.7, *P*=0.10) and 15 years (*t*=1.6, *P*=0.11), showing that the child's BMI did not affect whether the mother participated in the study at the follow-ups.

### Sample statistics

The mean BMI for mothers was 26.6 (s.d. 5.9). The characteristics of the child samples at the three follow-ups in this study are shown in Table 2.

### Aim 1: Describing and examining the mothers' classification of their child's weight status and concern about their child becoming overweight in the future

Table 3 shows that at each age, the proportion of mothers classifying a normal weight child as normal weight was high (over 99.0%). There was more variation across the ages in the proportion of mothers classifying an overweight child as overweight (36.2%, 52.3%, 39.2% at 7, 12 and 15 years, respectively). At each age, the majority of mothers with a normal weight child reported to be unconcerned about their child being overweight in the future. The majority of mothers with an overweight child did express concern about the child being overweight in the future; at 15 years, this level of concern was lower than at the other ages, but the proportions for concern were lower in overweight boys (64% compared with >79% at 7 and 12 years) than overweight girls (73% compared with >79% at 7 and 12 years;Table 3).

### Aim 2: Quantifying the weight at which mothers classify their child as overweight and express concern about their child becoming overweight in the future

Using logistic regression with the binary variable for mothers' classification of their child's weight status, at each age there was a statistically significant positive relationship between the mother classifying her child as overweight and the child's BMI (Table 4). Using the equations from the logistic regressions, for illustrative purposes Table 5 shows the BMI a child needs to be before half of mothers classify their child as overweight; at each age, both girls and boys need to be at the very top end of the overweight category or in the obese category (BMI centile range from 97 to 99) according to the UK 1990 growth reference curves.^2^

Similarly, at each age there were statistically significant positive relationships between the child's BMI and the mother expressing concern about her child becoming overweight in the future (Table 4). Interestingly, the point at which half of mothers expressed concern about their child's future weight was within the normal weight range or the lower level of the overweight category according to the UK 1990 growth reference curves,^2^ and this was lower for girls (BMI centile range from 75 to 91) than boys (BMI centile range from 81 to 94) (Table 5).

### Aim 3: Investigating characteristics associated with correct classification of child weight status

Logistic regression was used with the dichotomised correct/incorrect variable as the outcome. Table 6 shows that at 7, 12 and 15 years, child BMI was a significant predictor of a mother correctly classifying her child's weight status when adjusted for other variables. The mother correctly classifying her child's weight status at 12 years also predicted correctly classifying her child's weight status at 15 years. None of the remaining predictor variables (child's sex, pubertal maturation, mother's BMI or family economic status) were significant.

One important question to examine is whether there is a positive effect on the child's subsequent BMI if mothers do correctly classify their child as overweight at an earlier stage. For this reason, linear regression was used on the subsample of overweight or obese children at 7 years to examine whether the mothers' earlier perception of her child's weight status predicted later actual child BMI at 15 years (Table 7a), and similarly on the subsample of children overweight at 12 years (Table 7b). At both 7 and 12 years, child BMI strongly predicted BMI at 15 years but the child's weight gain was not changed by knowing whether the mother classified her child as overweight at an earlier stage. Further regressions were run to check whether this relationship was changed if the mother expressed concern about her child being overweight in the future, but including this variable did not change the pattern of statistical significance (data not shown).

## Discussion

This study found that the proportion of mothers with overweight children who classified their child as overweight did not substantially alter with child age. Our results clearly show that mothers at all three follow-ups were more likely to classify their overweight child as overweight as child BMI increased, but typically the child had to be at the higher end of the childhood overweight range or in the obese range before they described their child as overweight. Mothers expressed concern about their child becoming overweight in future at lower levels of BMI. Child's sex, pubertal maturation, mothers' BMI and family socioeconomic status were not related to the mothers' classification of their child's weight status. This study provides no evidence that mothers' correct classification of her child's overweight status at age 7 or 12 years is associated with relatively lower weight gain in her child either 8 or 3 years later, respectively.

Cross-sectional studies with a wide age range,^25, 26, 27^ one longitudinal study^15^ and a meta-analysis^4^ have shown that mothers are less likely to report their overweight child as overweight if the child is younger, particularly those under 6 years. In our study using a relatively large and representative population-based cohort, covering an 8-year age range from 7 years, we did not observe an age effect on mothers' correct classification of overweight in their child. Further longitudinal cohort studies will be needed to establish whether age effects exist or not. Similarly, the mothers' classification of the child's weight status was not found to be associated with the child's pubertal stage, which is supported by the only longitudinal study that has reported on pubertal development.^15^ The results from this study also showed no significant effects of mother's BMI or socioeconomic status, again supported by previous longitudinal studies.^15, 17^ The results on child's sex are more mixed; in our study, no sex effect was found, which is supported by one study^17^ but not another.^15^ Overall, these findings from the present study suggest that classifying a child's weight status, as might be expected, is based simply on the child's BMI; the only significant predictor of whether mothers classified their child as overweight was higher child BMI, also reported in other studies.^15, 16, 25, 26, 28^ This is a graded effect; mothers are more likely to classify their child as overweight if the child is obese rather than overweight.^8, 15, 29, 30^ The conclusion must be drawn that currently, using these methods, children need to be obese before mothers report them as being overweight.

The proportion of mothers who expressed concern about their overweight child being overweight in the future was lower at 15 years than at 7 and 12 years for overweight children, particularly for boys, and furthermore mothers expressed concern at a lower BMI UK 1990 centile in girls compared with boys. A plausible explanation for these results is that they reflect the higher expectation of boys being ‘big' compared with girls.^4^

There is an emphasis in the literature on the importance of the ability of mothers being able to identify correctly whether or not their child is overweight as this is regarded as key to parental action and intervention.^4, 5, 9, 10, 25, 31^ It is of particular note then that this longitudinal study, using repeated measures within the subset of children who are overweight, shows that this does not have a beneficial impact on later BMI—and this important result is consistent with those of a similar study of children over the same age range using the same methods.^15^ We do not know if the mothers in the current study who did classify their child as overweight took any weight management action; if they did it did not have a positive effect in terms of their child's BMI at a later stage. Mothers expressed concern about their child being overweight in future at much lower levels of current child BMI, but controlling for this in the analysis did not alter the results on later child BMI outcomes.

This study adds to the body of literature on maternal perceptions of child weight status. It reports on data from three time points using the same measures in a relatively large population-based cohort and the perceptions were assessed in narrow age ranges, which strengthens the ability to examine age-related effects. A limitation of this study is the fairly high level of attrition, and as is common for community-based studies this has been higher over time among the most deprived families.^32, 33^ However, due to over-representation of more deprived families at recruitment, the sample included in the analyses at the different time points have remained broadly representative of the north of England. It is possible that generalisability to other settings may be affected by the predominantly white ethnic background of the participants. In terms of examining maternal perceptions of their child's weight status and the child's weight gain, when interpreting the results it must be borne in mind that the samples for our weight gain analyses were small. Since this study was planned, three published articles report on this topic, showing that either children's weight gain is not relatively lower in the children whose mothers who reported their child as overweight at an earlier stage or that they gain more weight.^14, 15, 17^ Future studies need to be based on power calculations to further inform this field.

Research into maternal perceptions of childhood overweight proposes that mothers correctly classifying their child as overweight is a prerequisite for initiating successful action. The emerging evidence on outcomes for children in terms of their BMI brings this assumption into question. As the present study shows that parents tend to classify their child as overweight in only more extreme cases, one possible explanation for the finding that correct classification did not impact on later child BMI could be that these children are the most difficult for mothers to implement effective weight-related action. If mothers identified overweight in their child at a lower level, it is possible that they might be more able to take effective action to help their child return to a healthy body weight. Efforts are currently underway to raise parents' awareness of their child's weight status so that future studies will be in a position to examine this possibility.^34, 35^

## Figures and Tables

**Table 1:** Representativeness of original cohort and subsequent attrition (from Townsend deprivation index from data collected at birth)

	*Baseline (*N=*1011)*	*7 years (*N=*531)*	*12 years (*N=*433)*	*15 years (*N=*298)*
	n*(%)*	n*(%)*	n*(%)*	n*(%)*
*Townsend quintile*^a^				
1 (most affluent)	156 (15)	99 (19)	81 (19)	61 (20)
2	204 (20)	116 (22)	95 (22)	73 (25)
3	227 (23)	118 (22)	102 (24)	66 (22)
4	226 (22)	98 (18)	77 (18)	49 (16)
5 (least affluent)	192 (19)	94 (18)	73 (17)	46 (15)
Missing	6 (1)	6 (1)	5 (1)	3 (1)

Percentages do not add to 100 in all cases due to rounding.

a Based on Townsend deprivation index from 1991 UK census, using enumeration districts as the unit of analysis with the northern region of England as the population for comparison for the calculation of the quintiles.

**Table 2:** Descriptive statistics of samples of children at the three follow-ups

	*Boys*			*Girls*		
	*7 years*	*12 years*	*15 years*	*7 years*	*12 years*	*15 years*
N (%)	270 (50.8)	209 (48.3)	142 (47.7)	261 (49.2)	224 (51.7)	156 (52.3)
Age (years) (mean (s.d.))	7.4 (0.5)	12.5 (0.3)	15.2 (0.4)	7.5 (0.4)	12.5 (0.3)	15.2 (0.4)
						
*Pubertal maturation*^a^*(*n*(%))*						
Early			42 (30.2)			36 (23.2)
Mid			56 (40.3)			61 (39.4)
Late			41 (29.5)			58 (37.4)
BMI (mean (s.d.))	16.7 (2.4)	20.1 (3.8)	21.1 (4.2)	16.9 (2.4)	21.1 (4.0)	23.2 (4.7)
BMI SDS^b^ (mean (s.d.))	0.4 (1.2)	0.7 (1.3)	0.4 (1.3)	0.4 (1.0)	0.7 (1.1)	0.8 (1.2)
						
*Weight status*^b^ *(*n*(%))*						
Underweight	5 (1.9)	6 (2.9)	9 (6.3)	3 (1.1)	0 (0.0)	1 (0.6)
Normal	207 (76.7)	138 (66.0)	99 (69.7)	211 (80.8)	157 (70.1)	110 (70.5)
Overweight	33 (12.2)	37 (17.7)	15 (10.6)	31 (11.9)	42 (18.8)	26 (16.7)
Obese	25 (9.3)	28 (13.4)	19 (13.4)	16 (6.1)	25 (11.2)	19 (12.2)

Abbreviations: BMI, body mass index; N, number; SDS, standard deviation score.

Percentages do not add to 100 in all cases due to rounding.

a Missing for three boys and one girl.

b According to UK 1990 BMI reference curves for children.^2^

**Table 3:** Descriptive statistics of mothers' responses about child's weight category and concern about child being overweight in the future

*Mothers' response*	*Normal weight*^a^			*Overweight and obese*^a^		
	*7 years*	*12 years*	*15 years*	*7 years*	*12 years*	*15 years*
	n*(%)*	n*(%)*	n*(%)*	n*(%)*	n*(%)*	n*(%)*
*All*						
Not overweight	425 (99.8)	299 (99.3)	218 (99.5)	67 (63.8)	63 (47.7)	48 (60.8)
Overweight	1 (0.2)	2 (0.7)	1 (0.5)	38 (36.2)	69 (52.3)	31 (39.2)
						
*Boys*						
Not overweight	212 (100.0)	144 (100.0)	107 (99.1)	37 (63.8)	31 (47.7)	17 (50.0)
Overweight	0 (0.0)	0 (0.0)	1 (0.9)	21 (36.2)	34 (52.3)	17 (50.0)
						
*Girls*						
Not overweight	213 (99.5)	155 (98.7)	111 (100.0)	30 (63.8)	32 (47.8)	31 (68.9)
Overweight	1 (0.5)	2 (1.3)	0 (0.0)	17 (36.2)	35 (52.2)	14 (31.1)
						
*All*						
Not concerned	267 (62.7)	195 (64.8)	162 (74.0)	20 (19.0)	23 (17.4)	24 (30.4)
Concerned	159 (37.3)	106 (35.2)	57 (26.0)	85 (81.0)	109 (82.6)	55 (69.6)
						
*Boys*						
Not concerned	147 (69.3)	94 (65.3)	83 (76.9)	12 (20.7)	9 (13.8)	12 (35.3)
Concerned	65 (30.7)	50 (34.7)	25 (23.1)	46 (79.3)	56 (86.2)	22 (64.7)
						
*Girls*						
Not concerned	147 (69.3)	101 (64.3)	79 (71.2)	12 (20.7)	14 (20.9)	12 (26.7)
Concerned	65 (30.7)	56 (35.7)	32 (28.8)	46 (79.3)	53 (79.1)	33 (73.3)

a According to UK 1990 growth reference.^2^

**Table 4:** Logistic regressions predicting the likelihood of (a) the mother classifying her child as overweight and (b) expressing concern of her child becoming overweight in future, from child's BMI at 7, 12 and 15 years

	B	*S.e.*	P*-value*	*OR*	*95% CI*
*(a) Outcome variable: Classification of child's weight category (0=not overweight; 1=overweight)*					
Constant	−23.5				
7-year BMI^a^	1.1	0.1	<0.001	3.0	2.3–4.0
Constant	−24.6				
12-year BMI	1.0	0.1	<0.001	2.7	2.1–3.5
Constant	−16.5				
15-year BMI	0.6	0.1	<0.001	1.8	1.5–2.1
					
*(b) Outcome variable: Future concern of child becoming overweight (0=unconcerned; 1=concerned)*					
					
Constant	−7.7				
7-year BMI^a^	0.4	0.1	<0.001	1.6	1.4–1.7
Constant	−8.1				
12-year BMI	0.4	0.0	<0.001	1.5	1.4–1.6
Constant	−7.7				
15-year BMI	0.3	0.0	<0.001	1.4	1.3–1.5

Abbreviations: B, beta; BMI, body mass index; CI, confidence interval; OR, odds ratio*.*

*n*=531 at 7 years; *n*=433 at 12 years; *n*=298 at 15 years.

a Previously published data.^8^

**Table 5:** BMIs at which 50% of mothers (a) classify their children as currently overweight and (b) express concern that their children will become overweight in the future^a^

	*BMI*	*Associated UK 1990 centile*^*^2^*^	
		*Boys*	*Girls*
*(a) Classify their children as currently overweight*			
7 years^b^	23.1	99	99
12 years	24.4	99	97
15 years	29.1	99	99
			
*(b) Express concern that their children will become overweight in the future*			
7 years^b^	17.1	81	75
12 years	20.3	88	79
15 years	24.1	94	91

Abbreviation: BMI, body mass index.

a The proportions are derived from the logistic regression equations reported in Table 4.

b Previously published data.^8^

**Table 6:** Multiple logistic regressions predicting the likelihood of the mother correctly classifying her child's weight status^a^ at 7, 12 and 15 years

	*Mother correct at 7 years*		*Mother correct at 12 years*		*Mother correct at 15 years*	
	*OR (95% CI)*	P*-value*	*OR (95% CI)*	P*-value*	*OR (95% CI)*	P*-value*
Correct (12 years; yes)	—	—	—	—	7.0 (2.6–18.5)	<0.001
Correct (7 years; yes)	—	—	1.1 (0.4–2.8)	0.817	1.8 (0.6–5.6)	0.315
Child's sex (female)	1.2 (0.6–2.2)	0.577	1.4 (0.7–2.7)	0.322	0.8 (0.3–2.1)	0.692
Child's BMI (concurrent)	0.6 (0.5–0.7)	<0.001	0.8 (0.8–0.9)	<0.001	0.8 (0.7–0.9)	<0.001
Mother's BMI	1.0 (0.9–1.0)	0.368	1.0 (1.0–1.1)	0.327	1.1 (1.0–1.2)	0.149
Townsend deprivation index^b^	1.4 (0.9–1.4)	0.294	0.9 (0.8–1.1)	0.338	0.8 (0.6–1.2)	0.301
Pubertal maturation	—	—	—	—	1.7 (0.9–3.1)	0.084

Abbreviations: BMI, body mass index; CI, confidence interval; OR, odds ratio.

*n*=491 at 7 years; *n*=340 at 12 years; *n*=226 at 15 years.

a According to UK 1990 growth reference.^2^

b Based on 1991 UK census, using enumeration districts as the unit of analysis with the northern region of England as the population for comparison for the calculation of the quintiles.

**Table 7:** Multiple linear regression of factors (child BMI and maternal classification of child weight status) at 7 and 12 years predicting child BMI at 15 years

	*Unstandardised coefficients*		*Standardised coefficients*		
	B	*S.e.*	*Beta*	t*-value*	P*-value*
*(a) 7-Year predictors for children overweight at 7 years(*n=*50)*					
Constant	4.0				
Child's BMI (7 years)	1.1	0.4	0.5	3.1	0.003
Correct (7 years; yes)	0.3	1.7	0.0	0.2	0.842
					
*(b) 12-Year predictors for children overweight at 12 years(*n=*87)*					
Constant	−0.3				
Child's BMI (12 years)	1.1	0.1	0.8	9.0	<0.001
Correct (12 years; yes)	−0.2	0.8	0.0	−0.2	0.834

Abbreviations: B, unstandardised regression coefficient; beta, standardised regression coefficient; BMI, body mass index.

a According to UK 1990 growth reference.^2^
